# Changes in dispensing of medicines proposed for re-purposing in the first year of the COVID-19 pandemic in Australia

**DOI:** 10.1371/journal.pone.0269482

**Published:** 2022-06-15

**Authors:** Andrea L. Schaffer, David Henry, Helga Zoega, Julian H. Elliott, Sallie-Anne Pearson

**Affiliations:** 1 Centre for Big Data Research in Health, UNSW Sydney, Sydney, Australia; 2 Institute for Evidence-Based Healthcare, Bond University, Gold Coast, Australia; 3 Centre of Public Health Services, Faculty of Medicine, University of Iceland, Reykjavik, Iceland; 4 Cochrane Australia, School of Public Health and Preventive Medicine, Monash University, Monash, Australia; 5 Menzies Centre for Health Policy, The University of Sydney, Sydney, Australia; Seoul National University College of Medicine, REPUBLIC OF KOREA

## Abstract

**Background:**

Since COVID-19 was first recognised, there has been ever-changing evidence and misinformation around effective use of medicines. Understanding how pandemics impact on medicine use can help policymakers act quickly to prevent harm. We quantified changes in dispensing of common medicines proposed for “re-purposing” due to their perceived benefits as therapeutic or preventive for COVID-19 in Australia.

**Methods:**

We performed an interrupted time series analysis and cross-sectional study using nationwide dispensing claims data (January 2017-November 2020). We focused on six subsidized medicines proposed for re-purposing: hydroxychloroquine, azithromycin, ivermectin, colchicine, corticosteroids, and calcitriol (Vitamin D analog). We quantified changes in monthly dispensing and initiation trends during COVID-19 (March-November 2020) using autoregressive integrated moving average models and compared characteristics of initiators in 2020 and 2019.

**Results:**

In March 2020, we observed a 99% (95%CI: 96%-103%) increase in hydroxychloroquine dispensing (approximately 22% attributable to new users), and a 199% increase (95%CI: 184%-213%) in initiation, with an increase in prescribing by general practitioners (42% in 2020 vs 25% in 2019) rather than specialists. These increases subsided following regulatory restrictions on prescribing. There was a small but sustained increase in ivermectin dispensing over multiple months, with an 80% (95%CI 42%-118%) increase in initiation in May 2020 following its first identification as potentially disease-modifying in April. Other than increases in March related to stockpiling, we observed no change in the initiation of calcitriol or colchicine during COVID-19. Dispensing of corticosteroids and azithromycin was lower than expected from April through November 2020.

**Conclusions:**

While most increases in dispensing observed early on during COVID-19 were temporary and appear to be related to stockpiling among existing users, we observed increases in the initiation of hydroxychloroquine and ivermectin and a shift in prescribing patterns which may be related to the media hype around these medicines. A quick response by regulators can help limit inappropriate repurposing to lessen the impact on medicine supply and patient harm.

## Introduction

Since COVID-19 was first recognized, several medicines have been proposed for ‘re-purposing’ in the belief they may be effective in treating or preventing COVID-19, the disease caused by SARS-CoV-2 [1, 2]. Large, well-conducted trials have identified potential benefits for systemic corticosteroidsand cytokine inhibitors [3–6]. However, other medicines (e.g. hydroxychloroquine, ivermectin, Vitamin D) are not effective yet misinformation about their benefits has been widespread [6, 7]. There are reports of medicines being prescribed extensively despite a lack of robust efficacy or safety data [8]. For instance, in the United States (US) the number of new prescriptions for the antimalarial and anti-rheumatic medicine hydroxychloroquine was over 7-fold higher in March 2020 than in the previous year, with a shift in the characteristics of prescribers and people using the medicine [9].

Worldwide, the success of measures to reduce the spread of the virus has varied. In 2020, Australia had a relatively low incidence of COVID-19 compared with other countries, with a cumulative incidence of approximately 28,000 cases (109 per 100,000 population) and 908 deaths as of December 2020 [10]. Studies of changing medicine use patterns early on in the pandemic focussed on high COVID-19 incidence settings [9, 11]. The relatively low Australian infection rate means that substantial increases in the use of medicines believed to be of benefit in COVID-19 during this period are unlikely to be due to management of confirmed COVID-19; they more likely reflect stockpiling over concerns about supply shortages by people already using the medicine, or new use among people who believe in their preventive effect.

The early days of the COVID-19 pandemic were associated with great uncertainty; it is important to understand how large-scale health crises impact medicine use so that policymakers can act quickly to promote quality use of medicines and prevent harm. Given the ever-changing evidence, and some misinformation, around effective care of people with COVID-19, the Australian government established the National COVID-19 Clinical Evidence Taskforce, a multi-disciplinary collaboration between researchers and clinicians. Its role is to undertake continuous evidence surveillance and develop ‘living’ evidence-based guidelines, including recommendations for use of prescribed medicines in treating or preventing COVID-19 [12].

Our primary objective was to quantify changes in dispensing of hydroxychloroquine, azithromycin, ivermectin, colchicine, corticosteroids, and calcitriol, all widely available medicines in Australia that were proposed for re-purposing for prevention or treatment of COVID-19, in order to understand if and how use of these medicines changed in response to changing evidence and media attention. Our second objective was to quantify changes in initiation and patterns of use of these medicines, to determine if any observed increase in use was due to stockpiling among prevalent users concerned about supply shortages or new use among people who believed in their preventive or therapeutic effects for COVID-19.

## Materials and methods

### Context

Australia maintains a publicly funded, universal healthcare system entitling all citizens and eligible residents to subsidized prescribed medicines through the Pharmaceutical Benefits Scheme (PBS). Medicines dispensed by community pharmacies are known as the general schedule, or Section 85 (S85). In 2020, Australia had a low overall COVID-19 incidence but experienced two notable spikes in cases in March and July [10]. We selected March 2020 as the interruption point as this coincided with a nationwide emergency response plan and marked the start of the initial nationwide lockdown [10].

### Medicines of interest

We focused on six prescribed medicines available on the PBS general schedule (S85), that were proposed for re-purposing for prevention or treatment of COVID-19, most of which were the subject of extensive media coverage [13–15]. We used the National COVID-19 Clinical Evidence Taskforce’s Australian guidelines for the clinical care of people with COVID-19 [12] as of July 2021 to guide judgment about which of several categories applied: 1) recommended for use (corticosteroids), 2) should be used only in a clinical trial (ivermectin, calcitriol [Vitamin D analog]) or 3) should not be used for prevention or treatment of COVID-19 (hydroxychloroquine, azithromycin, colchicine) (S1 Table). We also looked at the use of hydroxychloroquine and azithromycin combined, which was reported early on as a potentially beneficial combination [2].

### Data sources

We used two sources of PBS data. First, we used publicly-available, monthly aggregate claims for all S85 medicines dispensed to PBS-eligible people from January 2017 to November 2020 to analyze overall changes in dispensing after March 2020 [16]. While these data capture all community dispensing in Australia, they do not contain person-level characteristics. For more detailed analyses, we used person-level claims for a 10% random sample of all PBS-eligible people for the same period. All Australian citizens and the majority of residents are PBS-eligible and during the study period these data captured medicine dispensing for approximately 1.7 million people per year. These data contain information on medicines dispensed, including prescriber specialty, and the patient’s year of birth and sex. The 10% sample is a standard dataset provided by Services Australia for analytical use and is selected based on the last digit of each person’s randomly assigned unique identifier. To protect privacy, dispensing dates are offset by +/-14 days; the offset is the same within each individual. PBS dispensing data mostly reflect prescribing in general practice with a small proportion from specialists in their offices, private hospital inpatients, and aged care residents. PBS claims do not capture medicines dispensed to public hospital inpatients or private dispensing (i.e., not PBS-subsidised where the consumer pays the entire cost out-of-pocket).

### Statistical analyses

We used the aggregated PBS data to quantify changes in all S85 medicines combined and each medicine of interest from March to November 2020. We used interrupted time series analyses with autoregressive integrated moving average (ARIMA) models to estimate monthly changes from March to November 2020 [17]. Details on the methodology are in the S1 Appendix. To account for stockpiling, we summed the change in the number of dispensings predicted by the model over all months to estimate the total change during the COVID-19 period. We estimated 95% confidence intervals (CIs) by summing lower and upper bounds of the estimated change in each month.

Second, using person-level data for 10% of PBS-eligible people, we examined patterns of dispensing and treatment initiation for each medicine of interest. We defined initiation as the first observed dispensing after 360 days without dispensing of that medicine. We performed interrupted time series analysis using ARIMA models as described above to quantify changes in the number of initiators. For medicines where we observed ≥1 month with a significant increase in initiation between March and November 2020, we compared the characteristics of people initiating (sex, age, prescriber specialty) to initiators during the same period in 2019.

In December 2019 and January 2020, Australia also experienced severe bushfires that may have impacted prescribed medicine use [18]. Therefore, we tested the inclusion of dummy variables representing this period in the modeling. As the impact was minimal, we removed the bushfire covariate from the final models.

We performed all analyses with R V4.0.2 and SAS V9.4.

### Ethics and data access approvals

This study was approved by the New South Wales Population and Health Services Research Ethics Committee (no. 2013/11/494). The Australian Government Services Australia External Request Evaluation Committee granted access to the 10% sample of PBS claims for the study (no. RMS1126).

## Results

### Overall dispensing

**Fig 1:**
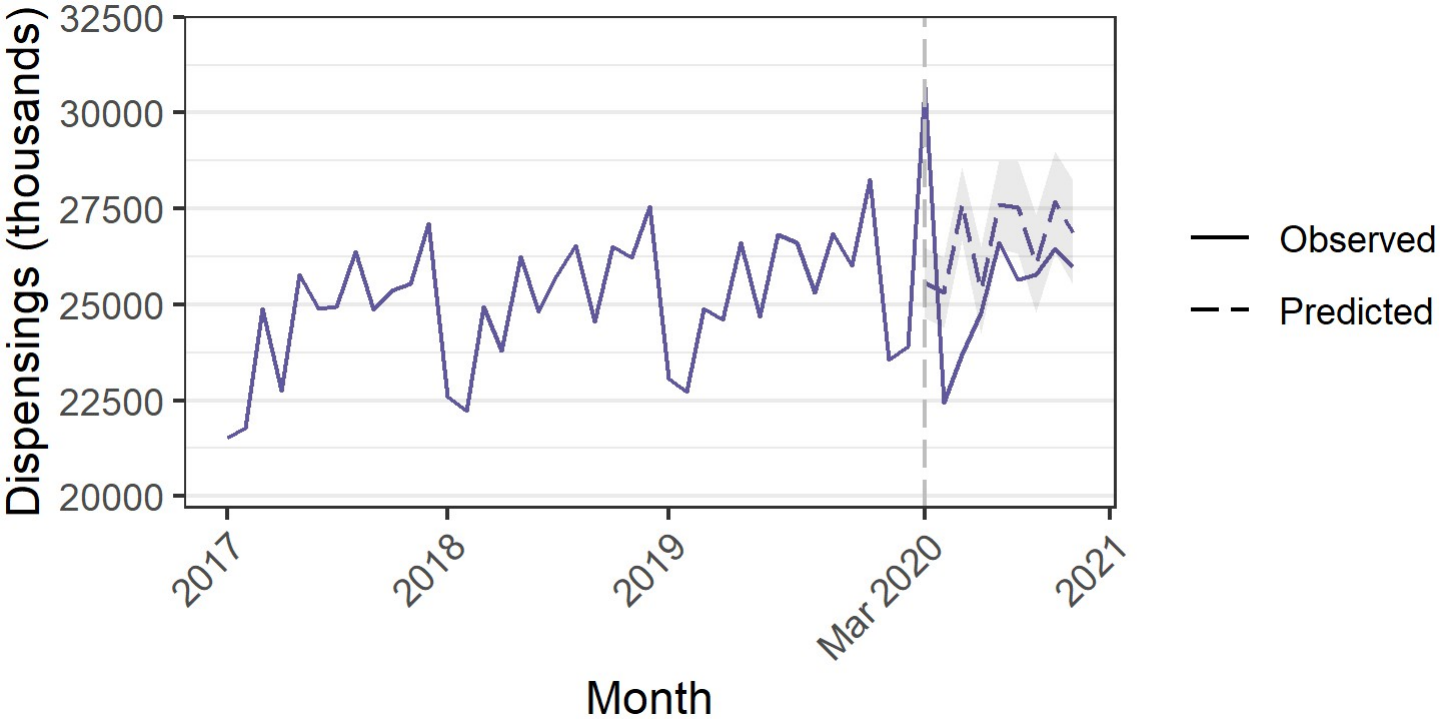
Dispensing of all medicines. Grey shaded area = 95% confidence interval for predicted values.

We observed a 20.0% (95%CI 17.0% to 23.0%) increase over the predicted estimates in all S85 PBS medicines dispensed in March 2020 of 5,110,790 (95%CI: 4,350,937 to 5,870,644). This was followed by several months of decreased dispensing (Fig 1, S2 Table).

### Azithromycin

**Fig 2:**
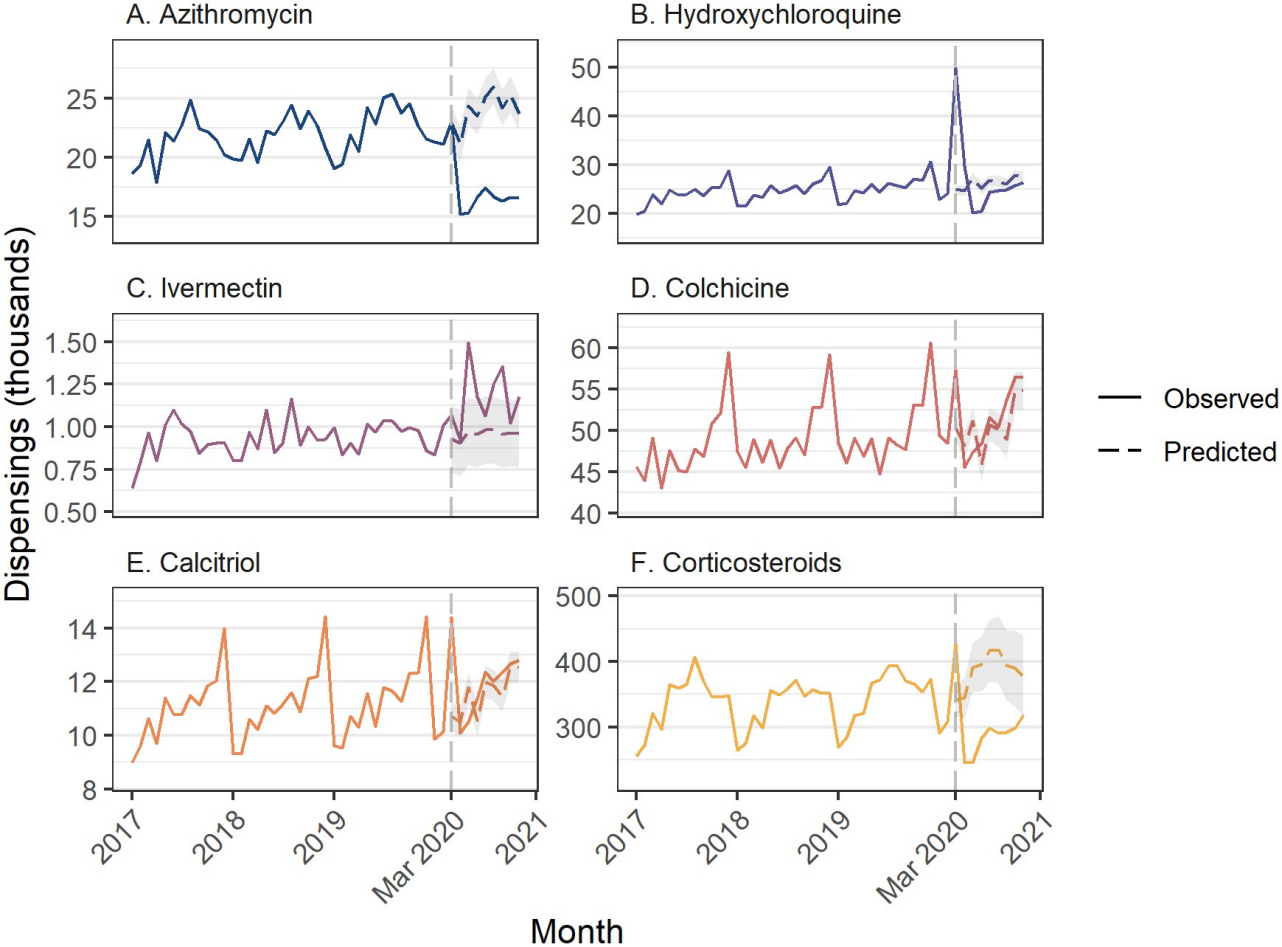
Dispensing of medicines of interest using full aggregate Section 85 dispensing data. Grey shaded area = 95% confidence interval for predicted values.

**Fig 3:**
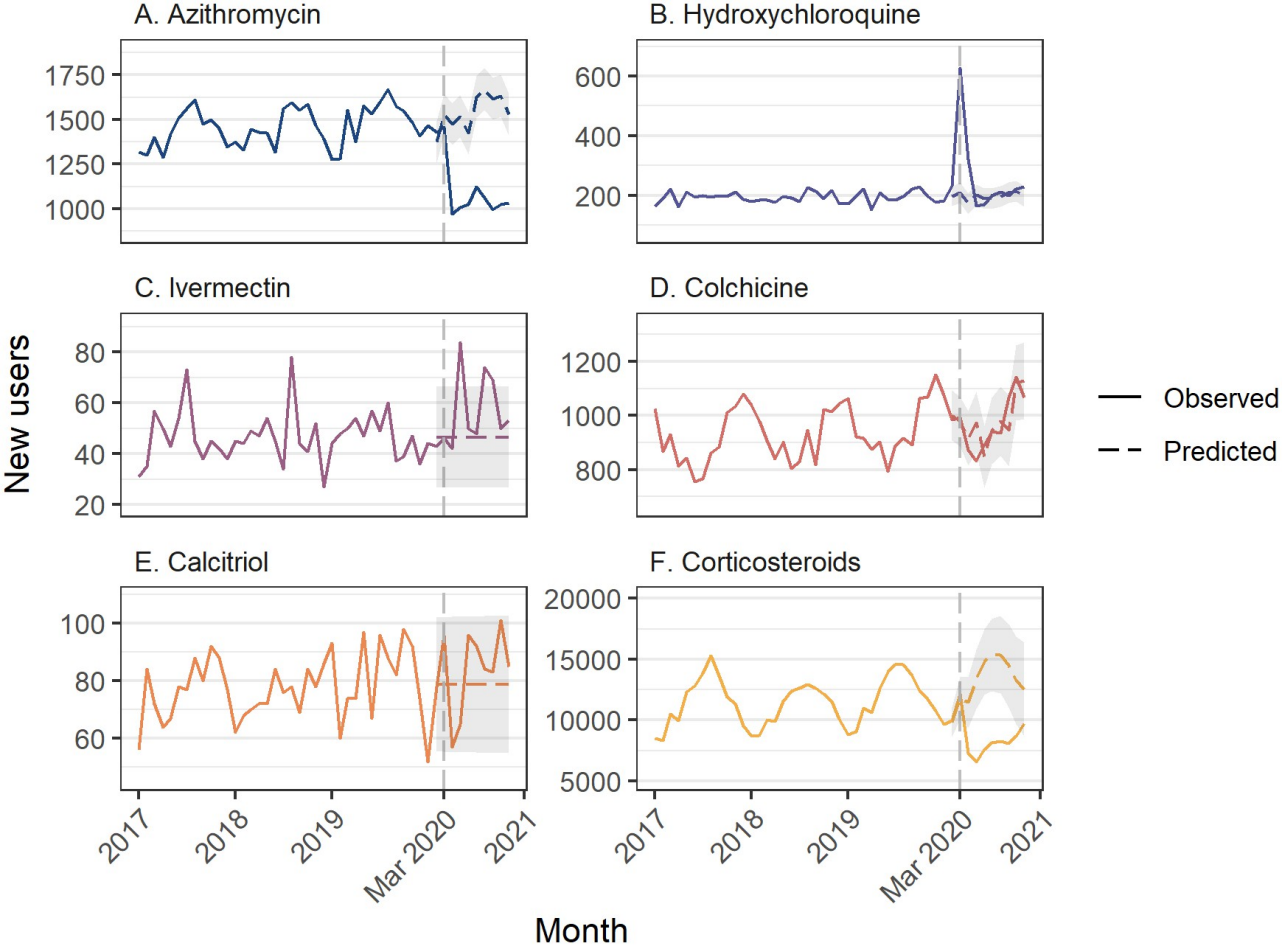
Number of new users of each medicine class of interest estimated using a 10% sample of PBS dispensing claims data. New user = new dispensing without a previous dispensing in the past 360 days. Grey shaded area = 95% confidence interval for predicted values.

We did not observe an increase in azithromycin dispensing in March 2020 (S2 Table). Azithromycin dispensing was lower than expected in April through November, and we observed 61,766 fewer dispensings during the COVID-19 period (95%CI: -72,457 to -51,075) (Fig 2A; Table 1). Similarly, in the PBS 10% sample data, we observed fewer people initiating azithromycin each month from April through November (Fig 3A; S3 Table).

**Table 1:** Change in dispensing from March to November 2020 estimated using autoregressive integrated moving average models (ARIMA) with full aggregate s85 dispensing data.

	Median monthly dispensing in 2019	Total change in dispensing, Mar-Nov 2020			
		N	95% CI	%	95% CI
**All dispensings**	25 666 813	-7 508 685	-15 967 327 to 949 957	-3.1%	-6.7% to 0.4%
Azithromycin	22 699	-61 766	-72 457 to -51 075	-28.6%	-33.5% to -23.6%
Hydroxychloroquine	25 481	9195	538 to 17 852	3.9%	0.2% to 7.5%
Ivermectin	977	1923	344 to 3501	22.3%	4.0% to 40.6%
Colchicine	48 767	12 230	-2510 to 26 969	2.7%	-0.6% to 5.9%
Corticosteroids	366 886	-767 236	-1 107 670 to -426 801	-22.1%	-31.9% to -12.3%
Calcitriol	11 413	4610	340 to 8879	4.4%	0.3% to 8.5%

CI = confidence interval

### Hydroxychloroquine

Prior to COVID-19, in 2019 the median monthly dispensing of hydroxychloroquine was 25,481 per month (Fig 2B). We observed an increase in hydroxychloroquine dispensing of 24,799 (95%CI: 23,887 to 25,711) in March 2020, representing a 99.4% increase (95%CI: 95.8% to 103.1%) over the predicted value (n = 24,944), and an increase of 4977 (95%CI: 4042 to 5912) in April. (S2 Table). While hydroxychloroquine dispensing was lower than predicted in May through October, it did not offset the earlier increases; there was an estimated 9195 more hydroxychloroquine dispensings (95%CI: 538 to 17,852) than predicted over the COVID-19 period (March through November) (Table 1).

In the 10% PBS sample, in 2019 we observed a monthly median of 2438 hydroxychloroquine dispensings and 281 people initiating. In March 2020, 415 additional people initiated hydroxychloroquine (95%CI: 385 to 446), a 198.6% increase (95%CI: 184.1% to 213.1%) (Fig 3B, S3 Table). In the 10% PBS sample we estimated that there were an additional 1884 dispensings in March 2020 (S4 Table), meaning that 78% of the spike in dispensing in March 2020 was among people previously treated with the medicine. Over the COVID-19 period, there were an additional 562 people who initiated hydroxychloroquine from March to November (95%CI 292 to 832) (Table 2).

**Table 2:** Change in number of initiators from March to November 2020 estimated using autoregressive integrated moving average models (ARIMA). Estimates are based on a 10% sample of PBS-eligible people.

	Median monthly initiators in 2019	Total change in initiation, Mar-Nov 2020			
		No. initiators	95% CI	%	95% CI
Azithromycin	1540	-4282	-5068 to -3496	-30.6%	-36.2% to -24.9%
Hydroxychloroquine	196	562	292 to 832	31.7%	16.4% to 46.9%
Ivermectin	48	97	-63 to 256	23.1%	-14.9% to 61.2%
Colchicine	916	-91	-974 to 792	-1.0%	-11.0% to 9.0%
Corticosteroids	12 080	-45 986	-67 768 to -24 204	-37.6%	-55.3% to -19.8%
Calcitriol	85	54	-135 to 242	7.6%	-19.1% to 34.2%

CI = confidence interval

We observed a significant increase in initiation of hydroxychloroquine therapy in March and April 2020, so we compared the characteristics of people initiating the medicine in these months with the same period in 2019. Of people with new hydroxychloroquine use in 2020, 30.1% were male compared with 23.4% during the same months in 2019 (Table 3). We observed a substantial increase in the number of new hydroxychloroquine dispensings written by GPs; in 2020, 41.8% (n = 533) were written by GPs compared with 24.6% (n = 129) in 2019. Conversely, the proportion of prescriptions written by rheumatologists was 45.3% (n = 238) in 2019 and 25.8% (n = 567) in 2020. Combined use of hydroxychloroquine and azithromycin was rare, with only 16 initiators of hydroxychloroquine also prescribed azithromycin in the same month during COVID-19.

**Table 3:** People initiating hydroxychloroquine and ivermectin in months in 2020 where there was a greater than expected number of new users, compared with new users in 2019. Estimates are based on a 10% sample of PBS dispensing claims data.

	Hydroxychloroquine		Ivermectin	
	Mar-Apr 2019	Mar-Apr 2020	May, Aug, Sept, Nov 2019	May, Aug, Sept, Nov 2020
No. dispensings	525	1276	202	536
**Sex**				
Female	402 (76.6)	892 (69.9)	102 (50.5)	277 (51.7)
Male	123 (23.4)	384 (30.1)	100 (49.5)	259 (48.3)
**Age group**				
<45 years	129 (24.6)	290 (22.7)	74 (36.6)	161 (30.0)
45–64 years	199 (37.9)	588 (46.1)	62 (30.7)	144 (26.9)
65–84 years	181 (34.5)	370 (29.0)	51 (25.3)	165 (30.8)
85+ years	16 (3.1)	28 (2.2)	15 (7.4)	66 (12.3)
**Prescriber specialty**				
GP	129 (24.6)	533 (41.8)	129 (63.9)	343 (64.0)
Rheumatology	238 (45.3)	567 (25.8)	N/A	N/A
Dermatology	N/A	N/A	26 (12.9)	70 (13.1)
Other	158 (30.1)	414 (32.5)	47 (23.3)	123 (23.0)

CI = confidence interval

### Ivermectin

We did not observe any increase in ivermectin dispensing in March 2020, but there was greater than expected ivermectin dispensing in May, June, August, September, and November 2020, with the largest increase in May 2020 (520, 95%CI 344 to 696, a 53.3% increase compared with predicted) (Fig 2C, S2 Table). Overall, there were 1923 more dispensings than expected during COVID-19 (95% CI: 344 to 3501) (Table 1).

In the PBS 10% sample, there was a monthly median of 90 ivermectin dispensings and 52 people initiating therapy in 2019. In May 2020 an additional 37 people initiated treatment (95%CI 20 to 55), an 80.4% increase (95%CI 42.3%-118.4%); however, there was no overall increase in the number of people initiating new treatment over the COVID-19 period (Fig 3C, Table 2, S3 Table).

As we observed a statistically significant increase in initiation of ivermectin in May, August, September, and November, we compared the characteristics of initiators in this period in 2020 with 2019. While the number of people initiating therapy was greater, there were few differences in their characteristics. In both 2019 and 2020, approximately 64% of new dispensings were prescribed by GPs.

### Colchicine

We observed an increase in colchicine dispensing in March of 7106 (95%CI 5814 to 8397) (Fig 2D, S1 Table). Smaller increases were also observed in June and September; however, there was no overall change in dispensing over the COVID-19 period (12,230, 95%CI -2510 to 26,969) (Table 1). We did not observe any months with an increase in people initiating colchicine treatment (S2 Table).

### Calcitriol (vitamin D analog)

We observed increases in calcitriol dispensing in March, June, and September 2020, with the greatest increase in March (3698, 95%CI 3256 to 4140, a 34.5% increase) (Fig 2E, S2 Table). In the 10% PBS sample data there was no change in the number of people initiating therapy in any month (Fig 2E, S3 Table).

### Corticosteroids

We observed fewer corticosteroid dispensings in all months from April through November (Fig 2F, S2 Table). Overall, there were 767,736 fewer dispensings than predicted (95%CI -1,107,670 to -426,801). In the 10% PBS sample, we observed a similar pattern with a decrease in initiation every month from April through October (Fig 3F, S3 Table), with no increase in any month.

## Discussion

During the COVID-19 epidemic in Australia as elsewhere, claims have been made about the unproven benefits of several common medicines. Australia provides a unique context for studying changes in the use of medicines proposed for repurposing for treatment or prevention of COVID-19 at the start of the pandemic due to the initial low incidence rate, meaning that the use of these medicines to treat people with confirmed cases of COVID-19 would be very rare. In this study, we found significant increases in dispensing of some of these medicines, but these trends were partly explained by stockpiling by people already on therapy, likely in anticipation of supply shortages and less access to medical practitioners.

As has been documented elsewhere [19, 20] dispensing of all PBS-listed medicines rose by approximately 20% in March 2020, followed by a decrease lasting several months, attributed to stockpiling by people receiving treatment for a broad range of disorders. Stockpiling has been observed internationally as well [21, 22]. While to our knowledge there have been no reports of people with legitimate needs for these medicines being unable to access them [23], nor increases in adverse events, stockpiling does put pressure on medicine supply and may exacerbate shortages. This is particularly true during crises such as the COVID-19 pandemic which led to disruptions of the global supply chain through lockdowns, understaffing, and travel bans [24]. While there are many causes for shortages, local regulatory agencies and policymakers can play a role in mitigating their short-term impacts [25, 26]. This may include identifying which medicines are most vulnerable to shortages, restricting the conditions under which they may be prescribed to limit waste and overuse, and acting quickly in response to changing circumstances [27, 28].

Hydroxychloroquine was one of the earliest PBS-listed medicine subjected to speculation in the media as a potential anti-viral treatment [8]. Hydroxychloroquine is used mainly as chronic treatment for autoimmune disorders and is ineffective in treating or preventing COVID-19; the Australian National Clinical Evidence Taskforce recommends against its use [29, 30] We observed a large spike in hydroxychloroquine dispensing early in the pandemic, with 78% driven by stockpiling by people already on treatment, some of whom may have been concerned by shortages. However, the greatest relative increase was related to new users, and more likely to have been prescribed by a GP rather than a specialist which is consistent with findings from the US, where a 10.5-fold increase in new prescriptions by primary care physicians was observed [9]. On March 24, due to concerns over off-label prescribing of hydroxychloroquine the Therapeutic Goods Administration (TGA) which regulates medicines in Australia, limited who could initiate therapy to relevant specialties [31] and following an update by the Taskforce that hydroxychloroquine was “not recommended” on April 30, the TGA further increased restrictions on prescribing [32]. We did not have data on prescribing indication but another Australian study found that only half of people newly prescribed hydroxychloroquine by GPs during COVID-19 had a relevant condition in their medical history [33].

Azithromycin, another treatment that received a ‘do not use’ recommendation by the Taskforce has been widely promoted as COVID-19 treatment as a sole therapy or in combination with hydroxychloroquine. There was no increase in dispensing at the start of the pandemic, but we observed a sustained fall through the remainder of the study period, a pattern also observed with corticosteroids; this has been noted for many antibiotics and is related to a reduction in respiratory infections during COVID-19 restrictions [34, 35]. We found few cases of co-dispensing of azithromycin and hydroxychloroquine among people initiating hydroxychloroquine, which is also consistent with previously reported general practice data [33].

In contrast to hydroxychloroquine, the spikes in the use of ivermectin, a widely promoted therapy whose use is currently not recommended outside trials by the National COVID-19 Clinical Evidence Taskforce nor the World Health Organisation [12, 36], occurred later in the pandemic and were more distributed across time. Its role as potentially disease-modifying in COVID-19 was not publicized until April 2020 [37, 38]. We did not observe any ivermectin stockpiling by people already on therapy, as it is typically taken to treat scabies as a one-off treatment. However, the changes we observed during the COVID-19 pandemic in Australia—an increase of around 1900 dispensings, on an annual background of 12,000—suggest a modest uptake in the belief that it can treat or prevent COVID-19. Ivermectin has continued to attract media attention with several recent clinical trials plagued by problems such as errors or fabrication [39]. Lastly, while vitamin D has been promoted as COVID-19 treatment it is not recommended by the Taskforce for use outside clinical trials. Our data were limited to dispensing of calcitriol, which did not exhibit much change in dispensing during the COVID-19 epidemic.

Our analysis focused on the first year of the pandemic and the recommendations were made based on best available evidence at the time; current advice may differ as more evidence has accumulated. Moreover, we now have a more nuanced understanding of the risks and benefits of pharmacotherapy and which subgroups may or may not benefit, such as people with high-risk comorbidities and pregnant women [40, 41]. The only medicine in our study currently recommended by the World Health Organisation living guidelines are systemic corticosteroids for people with severe COVID-19 only, with strong recommendations against use of either hydroxychloroquine or ivermectin [6].

### Strengths and limitations

We had complete capture of medicine dispensing for the whole Australian population, and person-level data on a 10% sub-sample. However, we do not have data on dispensing of private prescriptions, meaning we have likely underestimated the impact on the use of some medicines. We also did not have information on the indication for prescribing and cannot determine whether the use was off-label or related to COVID-19. However, given the very low incidence of COVID-19 during the study period, and that these data primarily represent community dispensing, it is likely that only a tiny minority of increases in the use of these medicines were for treatment of COVID-19, but more likely represent a response to media attention and/or stockpiling, as we observed changes in the pattern of use of some medicines (such as hydroxychloroquine) not consistent with typical use. Lastly, disruptions to medicine use during COVID-19 likely have multiple causes, including lockdown measures, changed interaction with the healthcare system, reduced circulation of respiratory and gastrointestinal infections, and fear of not being able to access medicines, and these cannot reliably be disentangled.

## Conclusions

We demonstrated temporary changes in dispensing of commonly used medicines that were proposed for re-purposing for the treatment and prevention of COVID-19 early in the pandemic, including a large short-lived increase in hydroxychloroquine dispensing, most of which may be due to anticipatory stockpiling, and a later smaller but longer-lasting increase in ivermectin dispensing. Balanced and informed communication of the changing evidence, including up-to-date and reliable access to evidence-informed advice, is necessary to minimize any negative health impacts related to the re-purposing of medicines. When similar situations arise, a quick response by regulators can help limit inappropriate re-purposing, to avoid supply shortages and potential harms.

## Supporting information

S1 Table List of pharmaceutical treatments reviewed by the National COVID-19 taskforce as of July 2021 and their availability in Australia. (PDF)

S2 Table Monthly change in dispensing in 2020 compared with predicted values estimated using ARIMA models with full aggregate Section 85 dispensing data. (PDF)

S3 Table Monthly change in new users in 2020 compared with predicted values estimated using ARIMA models with a 10% sample of PBS data where the dispensing date is offset by +/- 14 days. (PDF)

S4 Table Monthly change in dispensing in 2020 compared with predicted values estimated using ARIMA models with 10% sample of PBS data where the dispensing date is offset by +/- 14 days. (PDF)

S1 Appendix Autoregressive integrated moving average (ARIMA) modelling. (PDF)

S1 Data Minimal dataset underlying findings. (XLSX)
