# Supplementary material for: Changes in dispensing of medicines proposed for re-purposing in the first year of the COVID-19 pandemic in Australia
Source: PLoS One. 2022 Jun 15;17(6):e0269482. doi: 10.1371/journal.pone.0269482 (PMC9200317; doi:10.1371/journal.pone.0269482)
Supplement: S1 Table — (PDF) [file pone.0269482.s001.pdf]

S1 Table: List of pharmaceutical treatments reviewed by the National COVID-19 taskforce as of July 2021 and their availability in Australia

Medicine name	National COVID-19 Clinical Evidence Taskforce recommendation	Availability in Australia*
Azithromycin	Do not use	PBS-subsidised (S85)
Hydroxychloroquine	Do not use	PBS-subsidised (S85)
Ivermectin	Do not use outside clinical trials	PBS-subsidised (S85)
Colchicine	Do not use	PBS-subsidised (S85)
Corticosteroids	Use	PBS-subsidised (S85)
Calcitriol	Do not use outside clinical trials	PBS-subsidised (S85); other vitamin D analogues such as calcifediol are available over-the-counter

PBS = Pharmaceutical Benefits Scheme; S85 = Section 85 (Section 85 is the general schedule and refers to items dispensed by community pharmacies
