# Supplementary material for: Changes in dispensing of medicines proposed for re-purposing in the first year of the COVID-19 pandemic in Australia
Source: PLoS One. 2022 Jun 15;17(6):e0269482. doi: 10.1371/journal.pone.0269482 (PMC9200317; doi:10.1371/journal.pone.0269482)
Supplement: S2 Table — (PDF) [file pone.0269482.s002.pdf]

S2 Table. Monthly change in dispensing in 2020 compared with predicted values estimated using ARIMA models with full aggregate Section 85 dispensing data

Month in 2020	Azithromycin	Hydroxychloroquine	Ivermectin	Colchicine
	**Change in dispensings, n (95% CI)**	**Change in dispensings, n (95% CI)**	**Change in dispensings, n (95% CI)**	**Change in dispensings, n (95% CI)**
Mar	69 (-1033 to 1171)	24799 (23887 to 25711)	142 (-31 to 316)	7106 (5814 to 8397)
Apr	-5867 (-7042 to -4692)	4977 (4042 to 5912)	22 (-154 to 198)	-2640 (-3936 to -1344)
May	-8883 (-10142 to -7624)	-7032 (-8025 to -6038)	520 (344 to 696)	-3888 (-5232 to -2545)
Jun	-6748 (-7922 to -5575)	-4736 (-5742 to -3731)	221 (47 to 396)	2457 (799 to 4114)
Jul	-7533 (-8796 to -6269)	-2382 (-3379 to -1386)	80 (-97 to 257)	880 (-774 to 2533)
Aug	-9229 (-10404 to -8055)	-1898 (-2871 to -926)	263 (86 to 439)	231 (-1523 to 1984)
Sep	-7804 (-8995 to -6613)	-1287 (-2240 to -335)	399 (224 to 574)	4695 (2812 to 6578)
Oct	-8640 (-9808 to -7472)	-2048 (-3004 to -1091)	55 (-120 to 231)	1743 (-127 to 3613)
Nov	-7132 (-8316 to -5948)	-1197 (-2130 to -265)	220 (45 to 395)	1647 (-344 to 3638)

Month in 2020	Corticosteroids	Calcitriol	All medicines
	**Change in dispensings, n (95% CI)**	**Change in dispensings, n (95% CI)**	**Change in dispensings, n (95% CI)**
Mar	87195 (66643 to 107747)	3698 (3256 to 4140)	5110790 (4350937 to 5870644)
Apr	-98233 (-124238 to -72229)	-418 (-888 to 52)	-2865463 (-3623925 to -2107001)
May	-145302 (-176357 to -114246)	-1283 (-1756 to -810)	-3924526 (-4705203 to -3143850)
Jun	-112895 (-148176 to -77614)	830 (355 to 1305)	-582967 (-1531222 to 365288)
Jul	-117706 (-156781 to -78631)	367 (-110 to 844)	-1002106 (-1951755 to -52458)
Aug	-127310 (-169837 to -84783)	148 (-332 to 628)	-1881239 (-2871030 to -891448)
Sep	-102474 (-148194 to -56754)	866 (384 to 1348)	-268893 (-1339225 to 801440)
Oct	-90971 (-139675 to -42267)	139 (-345 to 623)	-1217241 (-2295849 to -138633)
Nov	-59540 (-111056 to -8025)	262 (-225 to 749)	-877041 (-2000055 to 245974)

ARIMA = autoregressive integrated moving average models
