# Supplementary material for: Changes in dispensing of medicines proposed for re-purposing in the first year of the COVID-19 pandemic in Australia
Source: PLoS One. 2022 Jun 15;17(6):e0269482. doi: 10.1371/journal.pone.0269482 (PMC9200317; doi:10.1371/journal.pone.0269482)
Supplement: S3 Table — (PDF) [file pone.0269482.s003.pdf]

S3 Table. Monthly change in new users in 2020 compared with predicted values estimated using ARIMA models with a 10% sample of PBS data where the dispensing date is offset by +/- 14 days

Month in 2020	Azithromycin	Hydroxychloroquine	Ivermectin	Colchicine
	**Change in dispensings, n (95% CI)**	**Change in dispensings, n (95% CI)**	**Change in dispensings, n (95% CI)**	**Change in dispensings, n (95% CI)**
Mar	-61 (-148 to 27)	415 (385 to 446)	-1 (-18 to 17)	14 (-64 to 91)
Apr	-510 (-597 to -423)	150 (119 to 182)	-5 (-22 to 13)	-42 (-126 to 42)
May	-500 (-588 to -411)	-37 (-67 to -8)	37 (20 to 55)	-142 (-235 to -50)
Jun	-380 (-471 to -290)	-21 (-51 to 8)	3 (-14 to 21)	46 (-47 to 138)
Jul	-503 (-589 to -416)	11 (-18 to 41)	1 (-16 to 19)	-1 (-101 to 100)
Aug	-603 (-690 to -516)	17 (-13 to 46)	27 (10 to 45)	-44 (-148 to 60)
Sep	-620 (-706 to -533)	-12 (-42 to 19)	22 (5 to 40)	121 (15 to 227)
Oct	-610 (-697 to -523)	6 (-25 to 37)	3 (-14 to 21)	21 (-91 to 132)
Nov	-496 (-583 to -410)	33 (3 to 62)	6 (-11 to 24)	-63 (-178 to 52)

Month in 2020	Corticosteroids	Calcitriol
	**Change in dispensings, n (95% CI)**	**Change in dispensings, n (95% CI)**
Mar	358 (-1077 to 1793)	18 (-13 to 49)
Apr	-4157 (-5914 to -2400)	-35 (-69 to -1)
May	-6778 (-8807 to -4749)	-11 (-47 to 25)
Jun	-7229 (-9498 to -4960)	26 (-12 to 64)
Jul	-7217 (-9702 to -4732)	18 (-22 to 58)
Aug	-7121 (-9805 to -4437)	-1 (-43 to 41)
Sep	-6411 (-9281 to -3541)	-5 (-49 to 39)
Oct	-4611 (-7655 to -1567)	46 (0 to 92)
Nov	-2820 (-6028 to 388)	21 (-26 to 68)

ARIMA = autoregressive integrated moving average models
