# Supplementary material for: Changes in dispensing of medicines proposed for re-purposing in the first year of the COVID-19 pandemic in Australia
Source: PLoS One. 2022 Jun 15;17(6):e0269482. doi: 10.1371/journal.pone.0269482 (PMC9200317; doi:10.1371/journal.pone.0269482)
Supplement: S4 Table — (PDF) [file pone.0269482.s004.pdf]

S4 Table. Monthly change in dispensing in 2020 compared with predicted values estimated using ARIMA models with 10% sample of PBS data where the dispensing date is offset by +/- 14 days

Month in 2020	Azithromycin	Hydroxychloroquine	Ivermectin	Colchicine
	Change in dispensings, n (95% CI)	Change in dispensings, n (95% CI)	Change in dispensings, n (95% CI)	Change in dispensings, n (95% CI)
Mar	89 (-53 to 231)	316 (222 to 411)	-7 (-34 to 20)	217 (96 to 337)
Apr	-28 (-231 to 174)	1884 (1785 to 1984)	-7 (-34 to 21)	479 (358 to 599)
May	-616 (-862 to -369)	750 (646 to 855)	-12 (-40 to 15)	-28 (-149 to 92)
Jun	-702 (-988 to -416)	-391 (-500 to -281)	48 (21 to 76)	-165 (-312 to -18)
Jul	-536 (-857 to -215)	-353 (-467 to -239)	23 (-5 to 50)	47 (-106 to 201)
Aug	-794 (-1142 to -446)	-184 (-302 to -65)	1 (-27 to 29)	188 (35 to 341)
Sep	-788 (-1164 to -412)	-115 (-238 to 8)	22 (-6 to 50)	-62 (-232 to 108)
Oct	-859 (-1260 to -457)	-109 (-236 to 18)	64 (36 to 91)	358 (189 to 527)
Nov	-931 (-1356 to -505)	-91 (-222 to 40)	23 (-5 to 50)	63 (-119 to 244)

Month in 2020	Corticosteroids	Calcitriol
	Change in dispensings, n (95% CI)	Change in dispensings, n (95% CI)
Mar	1787 (175 to 3399)	154 (53 to 256)
Apr	5686 (3406 to 7966)	263 (159 to 368)
May	-6983 (-9776 to -4190)	31 (-76 to 139)
Jun	-12010 (-15235 to -8785)	-68 (-178 to 41)
Jul	-11372 (-14977 to -7767)	91 (-22 to 203)
Aug	-10177 (-14126 to -6228)	22 (-93 to 138)
Sep	-11139 (-15405 to -6873)	36 (-81 to 154)
Oct	-9876 (-14436 to -5316)	41 (-79 to 162)
Nov	-7491 (-12328 to -2654)	111 (-11 to 234)

ARIMA = autoregressive integrated moving average models
