# Supplementary material for: Changes in dispensing of medicines proposed for re-purposing in the first year of the COVID-19 pandemic in Australia
Source: PLoS One. 2022 Jun 15;17(6):e0269482. doi: 10.1371/journal.pone.0269482 (PMC9200317; doi:10.1371/journal.pone.0269482)
Supplement: Attachment — Submitted filename: Response_to_Reviewers.pdf [file pone.0269482.s008.pdf]

14 March 2022

Dear Prof Yon,

Thank you for the opportunity to revise and resubmit our manuscript. We have addressed all journal, editor and reviewer comments below.

On behalf of all co-authors,

Dr Andrea Schaffer
Medicines Policy Research Unit
Centre for Big Data Research in Health
University of New South Wales
Sydney Australia

Journal requirements

1. When submitting your revision, we need you to address these additional requirements.

Please ensure that your manuscript meets PLOS ONE's style requirements, including those for file naming. The PLOS ONE style templates can be found at https://journals.plos.org/plosone/s/file?id=wjVg/PLOSOne_formatting_sample_main_body.pdf and https://journals.plos.org/plosone/s/file?id=ba62/PLOSOne_formatting_sample_title_authors_affiliations.pdf

We have modified the file to follow the style requirements.

2. This research is supported by the National Health and Medical Research Council (NHMRC, https://www.nhmrc.gov.au/) Centre of Research Excellence in Medicines Intelligence (#1196900). AS is supported by a NHMRC Early Career Fellowship (#1158763). HZ is supported by a University of New South Wales Scientia Fellowship (https://www.scientia.unsw.edu.au/). The funders had no role in study design, data collection and analysis, decision to publish, or preparation of the manuscript.

3. Thank you for stating in your Funding Statement:
(This research is supported by the National Health and Medical Research Council (NHMRC, https://www.nhmrc.gov.au/) Centre of Research Excellence in Medicines Intelligence (#1196900). AS is supported by a NHMRC Early Career Fellowship (#1158763). HZ is supported by a University of New South Wales Scientia Fellowship (https://www.scientia.unsw.edu.au/). The funders had no role in study design, data collection and analysis, decision to publish, or preparation of the manuscript.)

Please provide an amended statement that declares *all* the funding or sources of support (whether external or internal to your organization) received during this study, as detailed online in our guide for authors at http://journals.plos.org/plosone/s/submit-

now. Please also include the statement "There was no additional external funding received for this study." in your updated Funding Statement.
Please include your amended Funding Statement within your cover letter. We will change the online submission form on your behalf.

> We have provided our amended funding statement below. Note that the Scientia Program Award for A/Prof Zoega (HZ) does not have a grant number.
>
> *AS, SAP, DH and HZ are supported by the National Health and Medical Research Council (NHMRC, https://www.nhmrc.gov.au/) Centre of Research Excellence in Medicines Intelligence (#1196900). AS is supported by a NHMRC Early Career Fellowship (#1158763). HZ is supported by a University of New South Wales Scientia Program Award (https://www.scientia.unsw.edu.au/). The funders had no role in study design, data collection and analysis, decision to publish, or preparation of the manuscript. There was no additional external funding received for this study.*

4. In your Data Availability statement, you have not specified where the minimal data set underlying the results described in your manuscript can be found. PLOS defines a study's minimal data set as the underlying data used to reach the conclusions drawn in the manuscript and any additional data required to replicate the reported study findings in their entirety. All PLOS journals require that the minimal data set be made fully available. For more information about our data policy, please see http://journals.plos.org/plosone/s/data-availability.

Upon re-submitting your revised manuscript, please upload your study's minimal underlying data set as either Supporting Information files or to a stable, public repository and include the relevant URLs, DOIs, or accession numbers within your revised cover letter. For a list of acceptable repositories, please see http://journals.plos.org/plosone/s/data-availability#loc-recommended-repositories. Any potentially identifying patient information must be fully anonymized.

Important: If there are ethical or legal restrictions to sharing your data publicly, please explain these restrictions in detail. Please see our guidelines for more information on what we consider unacceptable restrictions to publicly sharing data: http://journals.plos.org/plosone/s/data-availability#loc-unacceptable-data-access-restrictions. Note that it is not acceptable for the authors to be the sole named individuals responsible for ensuring data access.

We will update your Data Availability statement to reflect the information you provide in your cover letter.

> The minimal dataset underlying the results in our study has been included as a supplementary file (S3).

5. Thank you for stating the following in the Acknowledgments Section of your manuscript: (This research is supported by the National Health and Medical Research Council (NHMRC) Centre of Research Excellence in Medicines Intelligence (#1196900). Dr Schaffer is supported by a NHMRC Early Career Fellowship (#1158763). A/Prof Zoega is supported by a UNSW Scientia Fellowship. We thank the Australian Government Services Australia for providing the data. Thank you to Prof Andrew Wilson for his input.)

We note that you have provided funding information that is not currently declared in your Funding Statement. However, funding information should not appear in the Acknowledgments section or other areas of your manuscript. We will only publish funding information present in the Funding Statement section of the online submission form.
Please remove any funding-related text from the manuscript and let us know how you would like to update your Funding Statement. Currently, your Funding Statement reads as follows:
(This research is supported by the National Health and Medical Research Council (NHMRC, https://www.nhmrc.gov.au/) Centre of Research Excellence in Medicines Intelligence (#1196900). AS is supported by a NHMRC Early Career Fellowship (#1158763). HZ is supported by a University of New South Wales Scientia Fellowship (https://www.scientia.unsw.edu.au/). The funders had no role in study design, data collection and analysis, decision to publish, or preparation of the manuscript.)
Please include your amended statements within your cover letter; we will change the online submission form on your behalf.

We have removed all funding related text from the Acknowledgments and provided an updated funding statement above.

6. Please include captions for your Supporting Information files at the end of your manuscript, and update any in-text citations to match accordingly. Please see our Supporting Information guidelines for more information: http://journals.plos.org/plosone/s/supporting-information.

We have modified the text as requested.

Editors' comments

# Please cite top-tier landmark papers.

A. Agarwal A et al. A living WHO guideline on drugs for covid-19. BMJ. 2020 Sep 4;370:m3379. doi: 10.1136/bmj.m3379. Update in: BMJ. 2020 Nov 19;371:m4475. Update in: BMJ. 2021 Mar 31;372:n860. Update in: BMJ. 2021 Jul 6;374:n1703. Update in: BMJ. 2021 Sep 23;374:n2219. PMID: 32887691.

B. Steroid part: Shin YH et al. Autoimmune inflammatory rheumatic diseases and COVID-19 outcomes in South Korea: a nationwide cohort study. Lancet Rheumatol. 2021 Oct;3(10):e698-e706. doi: 10.1016/S2665-9913(21)00151-X. Epub 2021 Jun 18. PMID: 34179832; PMCID: PMC8213376.

We have now cited these two papers. The first paper we cited in the Introduction, while we also cited both papers in the Discussion in the following paragraph:

"Our analysis focused on the first year of the pandemic and the recommendations were made based on best available evidence at the time; current advice may differ as more evidence has accumulated. Moreover, we now have a more nuanced understanding of the risks and benefits of these medicines, including in high risk subgroups, such as people with comorbidities and pregnant women.[40,41] The only medicine in our study currently recommended by the World Health Organisation living guidelines are systemic corticosteroids for people with severe COVID-19 only, with strong recommendations against use of either hydroxychloroquine or ivermectin.[6]"

<u>Reviewer 1</u>

Thank you to the reviewer for their comments.

1) Abstract
The "Background" part is missing in the abstract. The background is an important part of the structure of the abstract and I would recommend adding it. Without background it is not clear why this research is important, what is known in this topic and what is unknown, what triggered the authors to do this research.

> We have now added a Background to our abstract:
>
> *“Since COVID-19 was first recognised, there has been ever-changing evidence and misinformation around effective use of medicines. Understanding how pandemics impact on medicine use can help policymakers act quickly to prevent harm. We quantified changes in dispensing of common medicines proposed for “re-purposing” due to their perceived benefits as therapeutic or preventive for COVID-19 in Australia.”*

2) Introduction
Since I did not find a separate part in the draft of the manuscript that would be called "Objectives" and where the research objectives would be clearly and properly stated, I would recommend paying attention to the "Introduction" part, where the objectives of this research are stated at the end. I would recommend having a structured list of research objectives. Now they sound very similar and not clear. Also, I did not see the purpose for which the authors set the research objectives.
I would recommend having a primary objective, a secondary research objective, and at the end of each objective, add "in order to....."

> We have rewritten the last paragraph of the Introduction so that the objectives are clearer and more detailed:
>
> *“Our primary objective was to quantify changes in dispensing of hydroxychloroquine, azithromycin, ivermectin, colchicine, corticosteroids, and calcitriol, all widely available medicines in Australia that were proposed for re-purposing for prevention or treatment of COVID-19, in order to understand if and how use of these medicines changed in response to changing evidence and media attention. Our second objective was to quantify changes in initiation and patterns of use of these medicines, to determine if any observed increase in use was due to stockpiling among prevalent users concerned about supply shortages or new use among people who believed in their preventive or therapeutic effects for COVID-19.”*

3) Results
In this part, I noticed that in all tables and figures there is no footnote explaining 95%CI abbreviation. I would recommend adding a footnote to each table and figure that mentions the 95% CI abbreviation, so that the tables and figures are self-explanatory.

> We have now added this footnote to all relevant tables and figures.

<u>Reviewer 2</u>

Thank you to the reviewer for their feedback.

We have accepted all editorial and spelling/grammar changes made by the reviewer with tracked changes in the attached word document, with a couple of exceptions where we feel the edits changed the meaning of the text.

Note that we also duplicated the reviewer's comments from the word document below along with our response. The line numbers refer to the revised manuscript with tracked changes.

Line 194: What was the dispensing quantity before the study period? Please indicate these values to clearly show the stated increase in dispensing.

We have added the following sentence: "*Prior to the COVID-19 period, in 2019 the median dispensing of hydroxychloroquine was 25,481 per month*."

Line 197: Please indicate the predicted value

We have added this information in parentheses: *"…over the predicted value (n=24,944)*"

Line 202: How many people does the 10% PBS sample represent? Did you perform a normality test for the data? Why report median and not mean?

We have added information to the Methods on the composition of the 10% sample:

Line 121-24: *"For more detailed analyses, we used person-level claims for a 10% random sample of all PBS-eligible people for the same period. All Australian citizens and je majority of residents are PBS-eligible and during the study period these data capture medicine dispensing for approximately 1.7 million people per year."*

Regarding testing for normality, it is not considered useful with large sample sizes, as these tests will often detect even minor deviations from theoretical normality that are not of practical concern. Regardless, we have chosen to report the median as when the data are normally distributed, the mean and median will be the same (or very similar). However, when the data are not normally distributed, the median is more appropriate. Thus, there is no disadvantage in reporting the median instead of the mean, and it allowed us to report consistent measures across all medicine classes even if one or more was not normally distributed.

Line 206: Please this is a scientific report. Reword appropriately.

We have deleted the word "roughly". "*In the 10% PBS sample we estimated that there were an additional 1884 dispensings in March 2020 (Table S4), meaning that ~~roughly~~ 78% of the spike in dispensing in March 2020 was likely due to stockpiling among people previously treated with the medicine."*

Line 240: Was the increase statistically significant?

We have added the word "statistically" to the sentence. "*As we observed a <u>statistically</u> significant increase in initiation of ivermectin in May, August, September, and November….*"

Line 288: Please this is a scientific report. Reword appropriately.

We have reworded this to include the exact number*: "We observed a large spike in hydroxychloroquine dispensing early in the pandemic, with ~~roughly~~78% driven by stockpiling…"*
