# Supplementary material for: Changes in dispensing of medicines proposed for re-purposing in the first year of the COVID-19 pandemic in Australia
Source: PLoS One. 2022 Jun 15;17(6):e0269482. doi: 10.1371/journal.pone.0269482 (PMC9200317; doi:10.1371/journal.pone.0269482)
Supplement: Peer review history

# Peer review: Decision Letter 0

**DOI:** 10.1371/journal.pone.0269482.r001

**Authors:** Dong Keon Yon

12 Feb 2022

PONE-D-21-38759Changes in dispensing of medicines proposed for re-purposing in the first year of the COVID-19 pandemic in AustraliaPLOS ONE

Dear Dr. Schaffer,

Thank you for submitting your manuscript to PLOS ONE. After careful consideration, we feel that it has merit but does not fully meet PLOS ONE’s publication criteria as it currently stands. Therefore, we invite you to submit a revised version of the manuscript that addresses the points raised during the review process.

Please submit your revised manuscript by Mar 27 2022 11:59PM. If you will need more time than this to complete your revisions, please reply to this message or contact the journal office at plosone@plos.org. When you're ready to submit your revision, log on to https://www.editorialmanager.com/pone/ and select the 'Submissions Needing Revision' folder to locate your manuscript file.

Please include the following items when submitting your revised manuscript:

- A rebuttal letter that responds to each point raised by the academic editor and reviewer(s). You should upload this letter as a separate file labeled 'Response to Reviewers'.
- A marked-up copy of your manuscript that highlights changes made to the original version. You should upload this as a separate file labeled 'Revised Manuscript with Track Changes'.
- An unmarked version of your revised paper without tracked changes. You should upload this as a separate file labeled 'Manuscript'.

If you would like to make changes to your financial disclosure, please include your updated statement in your cover letter. Guidelines for resubmitting your figure files are available below the reviewer comments at the end of this letter.

If applicable, we recommend that you deposit your laboratory protocols in protocols.io to enhance the reproducibility of your results. Protocols.io assigns your protocol its own identifier (DOI) so that it can be cited independently in the future. For instructions see: https://journals.plos.org/plosone/s/submission-guidelines#loc-laboratory-protocols. Additionally, PLOS ONE offers an option for publishing peer-reviewed Lab Protocol articles, which describe protocols hosted on protocols.io. Read more information on sharing protocols at https://plos.org/protocols?utm_medium=editorial-email&utm_source=authorletters&utm_campaign=protocols.

We look forward to receiving your revised manuscript.

Kind regards,

Dong Keon Yon, MD, FACAAI

Academic Editor

PLOS ONE

Journal Requirements:

1. When submitting your revision, we need you to address these additional requirements.

Please ensure that your manuscript meets PLOS ONE's style requirements, including those for file naming. The PLOS ONE style templates can be found at 

https://journals.plos.org/plosone/s/file?id=wjVg/PLOSOne_formatting_sample_main_body.pdf and 

https://journals.plos.org/plosone/s/file?id=ba62/PLOSOne_formatting_sample_title_authors_affiliations.pdf

2.  This research is supported by the National Health and Medical Research Council (NHMRC, https://www.nhmrc.gov.au/) Centre of Research Excellence in Medicines Intelligence (#1196900). AS is supported by a NHMRC Early Career Fellowship (#1158763). HZ is supported by a University of New South Wales Scientia Fellowship (https://www.scientia.unsw.edu.au/). The funders had no role in study design, data collection and analysis, decision to publish, or preparation of the manuscript.

3. Thank you for stating in your Funding Statement: 

( This research is supported by the National Health and Medical Research Council (NHMRC, https://www.nhmrc.gov.au/) Centre of Research Excellence in Medicines Intelligence (#1196900). AS is supported by a NHMRC Early Career Fellowship (#1158763). HZ is supported by a University of New South Wales Scientia Fellowship (https://www.scientia.unsw.edu.au/). The funders had no role in study design, data collection and analysis, decision to publish, or preparation of the manuscript.)

Please provide an amended statement that declares *all* the funding or sources of support (whether external or internal to your organization) received during this study, as detailed online in our guide for authors at http://journals.plos.org/plosone/s/submit-now.  Please also include the statement “There was no additional external funding received for this study.” in your updated Funding Statement. 

Please include your amended Funding Statement within your cover letter. We will change the online submission form on your behalf.

4. In your Data Availability statement, you have not specified where the minimal data set underlying the results described in your manuscript can be found. PLOS defines a study's minimal data set as the underlying data used to reach the conclusions drawn in the manuscript and any additional data required to replicate the reported study findings in their entirety. All PLOS journals require that the minimal data set be made fully available. For more information about our data policy, please see http://journals.plos.org/plosone/s/data-availability.

Upon re-submitting your revised manuscript, please upload your study’s minimal underlying data set as either Supporting Information files or to a stable, public repository and include the relevant URLs, DOIs, or accession numbers within your revised cover letter. For a list of acceptable repositories, please see http://journals.plos.org/plosone/s/data-availability#loc-recommended-repositories. Any potentially identifying patient information must be fully anonymized.

Important: If there are ethical or legal restrictions to sharing your data publicly, please explain these restrictions in detail. Please see our guidelines for more information on what we consider unacceptable restrictions to publicly sharing data: http://journals.plos.org/plosone/s/data-availability#loc-unacceptable-data-access-restrictions. Note that it is not acceptable for the authors to be the sole named individuals responsible for ensuring data access.

We will update your Data Availability statement to reflect the information you provide in your cover letter.

5. Thank you for stating the following in the Acknowledgments Section of your manuscript: 

(This research is supported by the National Health and Medical Research Council (NHMRC) Centre of Research Excellence in Medicines Intelligence (#1196900). Dr Schaffer is supported by a NHMRC Early Career Fellowship (#1158763). A/Prof Zoega is supported by a UNSW Scientia Fellowship. We thank the Australian Government Services Australia for providing the data. Thank you to Prof Andrew Wilson for his input.)

We note that you have provided funding information that is not currently declared in your Funding Statement. However, funding information should not appear in the Acknowledgments section or other areas of your manuscript. We will only publish funding information present in the Funding Statement section of the online submission form. 

Please remove any funding-related text from the manuscript and let us know how you would like to update your Funding Statement. Currently, your Funding Statement reads as follows: 

( This research is supported by the National Health and Medical Research Council (NHMRC, https://www.nhmrc.gov.au/) Centre of Research Excellence in Medicines Intelligence (#1196900). AS is supported by a NHMRC Early Career Fellowship (#1158763). HZ is supported by a University of New South Wales Scientia Fellowship (https://www.scientia.unsw.edu.au/). The funders had no role in study design, data collection and analysis, decision to publish, or preparation of the manuscript.)

Please include your amended statements within your cover letter; we will change the online submission form on your behalf.

6. Please include captions for your Supporting Information files at the end of your manuscript, and update any in-text citations to match accordingly. Please see our Supporting Information guidelines for more information: http://journals.plos.org/plosone/s/supporting-information. 

Additional Editor Comments:

Thank you for submitting your manuscript to Plos One. The reviewers and I believe it is of potential value for our readers. However, the reviewers have raised a number of very important issues, and their excellent comments will need to be adequately addressed in a revision before the acceptability of your manuscript for publication in the Journal can be determined.

# Please cite top-tier landmark papers.

A. Agarwal A, Rochwerg B, Lamontagne F, Siemieniuk RA, Agoritsas T, Askie L, Lytvyn L, Leo YS, Macdonald H, Zeng L, Amin W, Barragan FAJ, Bausch FJ, Burhan E, Calfee CS, Cecconi M, Chanda D, Dat VQ, De Sutter A, Du B, Geduld H, Gee P, Harley N, Hashimi M, Hunt B, Jehan F, Kabra SK, Kanda S, Kim YJ, Kissoon N, Krishna S, Kuppalli K, Kwizera A, Lisboa T, Mahaka I, Manai H, Mino G, Nsutebu E, Preller J, Pshenichnaya N, Qadir N, Sabzwari S, Sarin R, Shankar-Hari M, Sharland M, Shen Y, Ranganathan SS, Souza JP, Stegemann M, Swanstrom R, Ugarte S, Venkatapuram S, Vuyiseka D, Wijewickrama A, Maguire B, Zeraatkar D, Bartoszko JJ, Ge L, Brignardello-Petersen R, Owen A, Guyatt G, Diaz J, Kawano-Dourado L, Jacobs M, Vandvik PO. A living WHO guideline on drugs for covid-19. BMJ. 2020 Sep 4;370:m3379. doi: 10.1136/bmj.m3379. Update in: BMJ. 2020 Nov 19;371:m4475. Update in: BMJ. 2021 Mar 31;372:n860. Update in: BMJ. 2021 Jul 6;374:n1703. Update in: BMJ. 2021 Sep 23;374:n2219. PMID: 32887691.

B. Steroid part: Shin YH, Shin JI, Moon SY, Jin HY, Kim SY, Yang JM, Cho SH, Kim S, Lee M, Park Y, Kim MS, Won HH, Hong SH, Kronbichler A, Koyanagi A, Jacob L, Smith L, Lee KH, Suh DI, Lee SW, Yon DK. Autoimmune inflammatory rheumatic diseases and COVID-19 outcomes in South Korea: a nationwide cohort study. Lancet Rheumatol. 2021 Oct;3(10):e698-e706. doi: 10.1016/S2665-9913(21)00151-X. Epub 2021 Jun 18. PMID: 34179832; PMCID: PMC8213376.

[Note: HTML markup is below. Please do not edit.]

Reviewers' comments:

Reviewer's Responses to Questions

**Comments to the Author**

1. Is the manuscript technically sound, and do the data support the conclusions?

The manuscript must describe a technically sound piece of scientific research with data that supports the conclusions. Experiments must have been conducted rigorously, with appropriate controls, replication, and sample sizes. The conclusions must be drawn appropriately based on the data presented. 

Reviewer #1: Yes

Reviewer #2: Partly

**********

2. Has the statistical analysis been performed appropriately and rigorously? 

Reviewer #1: Yes

Reviewer #2: No

**********

3. Have the authors made all data underlying the findings in their manuscript fully available?

The PLOS Data policy requires authors to make all data underlying the findings described in their manuscript fully available without restriction, with rare exception (please refer to the Data Availability Statement in the manuscript PDF file). The data should be provided as part of the manuscript or its supporting information, or deposited to a public repository. For example, in addition to summary statistics, the data points behind means, medians and variance measures should be available. If there are restrictions on publicly sharing data—e.g. participant privacy or use of data from a third party—those must be specified.

Reviewer #1: Yes

Reviewer #2: Yes

**********

4. Is the manuscript presented in an intelligible fashion and written in standard English?

PLOS ONE does not copyedit accepted manuscripts, so the language in submitted articles must be clear, correct, and unambiguous. Any typographical or grammatical errors should be corrected at revision, so please note any specific errors here.

Reviewer #1: Yes

Reviewer #2: Yes

**********

5. Review Comments to the Author

Please use the space provided to explain your answers to the questions above. You may also include additional comments for the author, including concerns about dual publication, research ethics, or publication ethics. (Please upload your review as an attachment if it exceeds 20,000 characters)

Reviewer #1: 1) Abstract

The "Background" part is missing in the abstract. The background is an important part of the structure of the abstract and I would recommend adding it. Without background it is not clear why this research is important, what is known in this topic and what is unknown, what triggered the authors to do this research.

2) Introduction

Since I did not find a separate part in the draft of the manuscript that would be called "Objectives" and where the research objectives would be clearly and properly stated, I would recommend paying attention to the "Introduction" part, where the objectives of this research are stated at the end. I would recommend having a structured list of research objectives. Now they sound very similar and not clear. Also, I did not see the purpose for which the authors set the research objectives.

I would recommend having a primary objective, a secondary research objective, and at the end of each objective, add "in order to....."

3) Results

In this part, I noticed that in all tables and figures there is no footnote explaining 95%CI abbreviation. I would recommend adding a footnote to each table and figure that mentions the 95% CI abbreviation, so that the tables and figures are self-explanatory.

Reviewer #2: The manuscript is well written and has potential.

However, some technical and typographical errors have been noted and highlighted. Please also note that full-stops come after cited references within the main text.

The attached document contains the comments that needs to be addressed.

Good job

**********

6. PLOS authors have the option to publish the peer review history of their article (what does this mean?). If published, this will include your full peer review and any attached files.

If you choose “no”, your identity will remain anonymous but your review may still be made public.

**Do you want your identity to be public for this peer review?** For information about this choice, including consent withdrawal, please see our Privacy Policy.

Reviewer #1: **Yes: **Dr. Oksana Artemchuk

Reviewer #2: No

[NOTE: If reviewer comments were submitted as an attachment file, they will be attached to this email and accessible via the submission site. Please log into your account, locate the manuscript record, and check for the action link "View Attachments". If this link does not appear, there are no attachment files.]

While revising your submission, please upload your figure files to the Preflight Analysis and Conversion Engine (PACE) digital diagnostic tool, https://pacev2.apexcovantage.com/. PACE helps ensure that figures meet PLOS requirements. To use PACE, you must first register as a user. Registration is free. Then, login and navigate to the UPLOAD tab, where you will find detailed instructions on how to use the tool. If you encounter any issues or have any questions when using PACE, please email PLOS at figures@plos.org. Please note that Supporting Information files do not need this step.

Attachment Submitted filename: COVID_Manuscript_PLOSONE.docx Click here for additional data file.

# Author comment: Author response to Decision Letter 0

**DOI:** 10.1371/journal.pone.0269482.r002

22 Mar 2022

Our response to the reviewer and editor comments are included in the attached document ("Response_to_Reviewers.pdf").

Attachment Submitted filename: Response_to_Reviewers.pdf Click here for additional data file.

# Peer review: Decision Letter 1

**DOI:** 10.1371/journal.pone.0269482.r003

**Authors:** Dong Keon Yon

23 May 2022

Changes in dispensing of medicines proposed for re-purposing in the first year of the COVID-19 pandemic in Australia

PONE-D-21-38759R1

Dear Dr. Schaffer,

We’re pleased to inform you that your manuscript has been judged scientifically suitable for publication and will be formally accepted for publication once it meets all outstanding technical requirements.

Within one week, you’ll receive an e-mail detailing the required amendments. When these have been addressed, you’ll receive a formal acceptance letter and your manuscript will be scheduled for publication.

An invoice for payment will follow shortly after the formal acceptance. To ensure an efficient process, please log into Editorial Manager at http://www.editorialmanager.com/pone/, click the 'Update My Information' link at the top of the page, and double check that your user information is up-to-date. If you have any billing related questions, please contact our Author Billing department directly at authorbilling@plos.org.

If your institution or institutions have a press office, please notify them about your upcoming paper to help maximize its impact. If they’ll be preparing press materials, please inform our press team as soon as possible -- no later than 48 hours after receiving the formal acceptance. Your manuscript will remain under strict press embargo until 2 pm Eastern Time on the date of publication. For more information, please contact onepress@plos.org.

Kind regards,

Dong Keon Yon, MD, FACAAI

Academic Editor

PLOS ONE

Additional Editor Comments (optional):

I congratulate the authors on this mermerzing work.

Reviewers' comments:

Reviewer's Responses to Questions

**Comments to the Author**

1. If the authors have adequately addressed your comments raised in a previous round of review and you feel that this manuscript is now acceptable for publication, you may indicate that here to bypass the “Comments to the Author” section, enter your conflict of interest statement in the “Confidential to Editor” section, and submit your "Accept" recommendation.

Reviewer #1: All comments have been addressed

**********

2. Is the manuscript technically sound, and do the data support the conclusions?

The manuscript must describe a technically sound piece of scientific research with data that supports the conclusions. Experiments must have been conducted rigorously, with appropriate controls, replication, and sample sizes. The conclusions must be drawn appropriately based on the data presented. 

Reviewer #1: Yes

**********

3. Has the statistical analysis been performed appropriately and rigorously? 

Reviewer #1: Yes

**********

4. Have the authors made all data underlying the findings in their manuscript fully available?

The PLOS Data policy requires authors to make all data underlying the findings described in their manuscript fully available without restriction, with rare exception (please refer to the Data Availability Statement in the manuscript PDF file). The data should be provided as part of the manuscript or its supporting information, or deposited to a public repository. For example, in addition to summary statistics, the data points behind means, medians and variance measures should be available. If there are restrictions on publicly sharing data—e.g. participant privacy or use of data from a third party—those must be specified.

Reviewer #1: Yes

**********

5. Is the manuscript presented in an intelligible fashion and written in standard English?

PLOS ONE does not copyedit accepted manuscripts, so the language in submitted articles must be clear, correct, and unambiguous. Any typographical or grammatical errors should be corrected at revision, so please note any specific errors here.

Reviewer #1: Yes

**********

6. Review Comments to the Author

Please use the space provided to explain your answers to the questions above. You may also include additional comments for the author, including concerns about dual publication, research ethics, or publication ethics. (Please upload your review as an attachment if it exceeds 20,000 characters)

Reviewer #1: (No Response)

**********

7. PLOS authors have the option to publish the peer review history of their article (what does this mean?). If published, this will include your full peer review and any attached files.

If you choose “no”, your identity will remain anonymous but your review may still be made public.

**Do you want your identity to be public for this peer review?** For information about this choice, including consent withdrawal, please see our Privacy Policy.

Reviewer #1: No

# Editor report: Acceptance letter

**DOI:** 10.1371/journal.pone.0269482.r004

**Authors:** Dong Keon Yon

31 May 2022

PONE-D-21-38759R1 

Changes in dispensing of medicines proposed for re-purposing in the first year of the COVID-19 pandemic in Australia 

Dear Dr. Schaffer:

I'm pleased to inform you that your manuscript has been deemed suitable for publication in PLOS ONE. Congratulations! Your manuscript is now with our production department. 

If your institution or institutions have a press office, please let them know about your upcoming paper now to help maximize its impact. If they'll be preparing press materials, please inform our press team within the next 48 hours. Your manuscript will remain under strict press embargo until 2 pm Eastern Time on the date of publication. For more information please contact onepress@plos.org.

If we can help with anything else, please email us at plosone@plos.org. 

Thank you for submitting your work to PLOS ONE and supporting open access. 

Kind regards, 

PLOS ONE Editorial Office Staff

on behalf of

Dr. Dong Keon Yon 

Academic Editor

PLOS ONE
